# Os Pacientes de Baixo Risco Cardiovascular Poderiam Ser Melhor Estratificados?

**DOI:** 10.36660/abc.20230310

**Published:** 2023-06-28

**Authors:** Francisco Antonio Helfenstein Fonseca, Maria Cristina Izar

**Affiliations:** 1 Escola Paulista de Medicina Universidade Federal de São Paulo São Paulo SP Brasil Escola Paulista de Medicina da Universidade Federal de São Paulo, São Paulo, SP – Brasil; 2 Universidade Federal de São Paulo São Paulo SP Brasil Universidade Federal de São Paulo – Medicina, São Paulo, SP – Brasil

**Keywords:** Baixo Risco Cardiovascular, Prevenção, DCVAS

Nesta edição dos Arquivos Brasileiros de Cardiologia, Cesena et al.^1^ determinaram a distribuição de percentis do risco de doença cardiovascular aterosclerótica (DCVAS) em 10 anos de acordo com idade e sexo, em uma grande população brasileira, com base nas equações de coorte agrupadas ACC/AHA.^1^ Eles excluíram pacientes com risco cardiovascular alto ou muito alto (por exemplo, aqueles com DCVAS conhecida, diabetes, hipercolesterolemia grave, doença renal crônica ou pessoas com idades fora da faixa de 40 a 75 anos). Os autores encontraram muitos indivíduos com baixo risco cardiovascular, mas acima do percentil 75 de risco de DCVAS para essa categoria. Esses achados fascinantes mostram as limitações da estimativa atual de risco cardiovascular em 10 anos, principalmente entre pacientes jovens. De fato, os autores apresentam uma ferramenta que pode ser muito útil para uma melhor sensibilização para o risco cardiovascular, abrindo a perspectiva para uma mudança precoce do estilo de vida ou para o início de terapêutica farmacológica.

Atualmente, muitos pacientes recebem terapia farmacológica ou mudam seu estilo de vida apenas no estágio avançado da doença cardiovascular.^2 , 3^ Nesse ponto, o cuidado cardiovascular é muito mais especializado e caro, e a qualidade de vida muitas vezes permanece comprometida apesar de grandes esforços.

Nos últimos anos, Fuster e Braunwald, considerados grandes referências da cardiologia moderna, propuseram a prevenção primordial das doenças cardiovasculares, evitando o desenvolvimento de fatores de risco ou a progressão da aterosclerose.^4 , 5^

A principal causa de morte e incapacidade em todo o mundo continua sendo a DCVAS, especialmente o infarto do miocárdio. No entanto, independentemente de outros fatores de risco cardiovascular (por exemplo, hipertensão, diabetes ou tabagismo), a exposição a níveis mais baixos de colesterol ao longo da vida reduz drasticamente a incidência de doença cardíaca coronária.^6^

Assim, em harmonia com o futuro da cardiologia moderna, Cesena et al.^1^ propõem uma abordagem que permite uma avaliação do risco cardiovascular futuro de nossos pacientes individuais. Neste momento, terapias menos intensivas podem ser implementadas, proporcionando melhor tolerabilidade e a adesão dos nossos pacientes.^1^

De acordo com o estudo de Cesena et al.,^1^ as principais características de baixo risco para DCVAS, mas acima do percentil 75 são, geralmente, pacientes com sobrepeso ou obesidade com dislipidemia e/ou tabagismo. Assim, os componentes da síndrome metabólica que não são contabilizados nas calculadoras de risco foram frequentemente identificados nesses pacientes, e todos esses fatores de risco podem ser abordados para uma diminuição a longo prazo do risco cardiovascular.^1^

Outro aspecto importante dessa contribuição é a estimativa de risco sem imagem. Nesse contexto, o escore de cálcio coronariano tem sido proposto para melhor estratificação dos pacientes de risco intermediário pelas diretrizes.^7^ O risco percentual também pode ser útil para identificar aqueles pacientes que não precisam ser encaminhados para análise de imagem, diminuindo custos.

Finalmente, o uso de percentis para risco de DCVAS de acordo com sexo, idade, e a raça foi previamente determinado na população dos EUA.^8^ Curiosamente, algumas diferenças nos percentis 25, 50 e 75 foram encontradas, mostrando que não podemos extrapolar dados de uma população para outra. Portanto, iniciativas para estabelecer o risco de DCVAS em percentis de nossa própria população parecem realmente importantes.
