# Supplementary material for: Os Pacientes de Baixo Risco Cardiovascular Poderiam Ser Melhor Estratificados?
Source: Arq Bras Cardiol. 2023 Jun 28;120(6):e20230310. [Article in Portuguese] doi: 10.36660/abc.20230310 (PMC10364996; doi:10.36660/abc.20230310)
Supplement: Translation

# Translation: Could Patients At Low Cardiovascular Risk Be Better Stratified?

**Authors:** Francisco Antonio Helfenstein Fonseca, Maria Cristina Izar

**Affiliations:** 1 Brazil Escola Paulista de Medicina da Universidade Federal de São Paulo, São Paulo, SP – Brazil; 2 Brazil Universidade Federal de São Paulo – Medicina, São Paulo, SP – Brazil

**Keywords:** Low Cardiovascular Risk, Prevention: ASCVD

In this issue of the *Arquivos Brasileiros de Cardiologia* , Cesena et al.^1^ determined the percentiles distribution of the 10-year Aatherosclerotic cardiovascular disease (ASCVD) risk according to age and gender, in a large Brazilian population, based on the ACC/AHA pooled cohort equations.^1^ They excluded patients at high or very high cardiovascular risk (e.g., those with known ASCVD, diabetes, severe hypercholesterolemia, chronic kidney disease, or people with ages outside the range of 40-75 years). The authors found many individuals at low cardiovascular risk but above the 75^th^percentile of ASCVD risk for that category. These fascinating findings show the limitations of the current estimation of 10-year cardiovascular risk, mainly among young patients. In fact, the authors present a tool that can be very useful for better awareness of cardiovascular risk, opening the perspective for an early change in lifestyle or for starting pharmacological therapy.

Currently, many patients receive pharmacological therapy or change their lifestyle only at the advanced stage of cardiovascular disease.^2 , 3^ At this point, cardiovascular care is much more specialized and expensive, and the quality of life often remains compromised despite great efforts.

In recent years, Fuster and Braunwald, considered major references in modern cardiology, have proposed the primordial prevention of cardiovascular disease, avoiding the development of risk factors or the progression of atherosclerosis.^4 , 5^

The leading cause of death and disability worldwide remains ASCVD, especially myocardial infarction. However, independently of other cardiovascular risk factors (e.g., hypertension, diabetes, or smoking), exposure to lower cholesterol levels over the lifespan dramatically reduces the incidence of coronary heart disease.^6^

Thus, in harmony with the future of modern cardiology, Cesena et al.^1^ propose an approach that allows an assessment of the future cardiovascular risk of our individual patients. At this time, less intensive therapies can be implemented, providing better tolerability and adherence for our patients.^1^

According to the study of Cesena et al.^1^ study, the main characteristics of low ASVCD cardiovascular risk but above the 75^th^percentile are generally overweight or obese patients with dyslipidemia and/or smokers. Thus, the components of metabolic syndrome that are not accounted for in risk calculators were frequently identified in these patients, and all these risk factors could be addressed for a long-term decrease in cardiovascular risk.^1^

Another important aspect of this contribution is the risk estimation without imaging. In this context, coronary calcium score has been proposed for better stratification of patients at intermediate risk by guidelines.^7^ The percentile risk can also be useful to identify those patients that do not need to be referred for imaging analysis, decreasing costs.

Finally, the use of percentiles for ASCVD risk according to sex, age, and race was previously determined in the USA population.^8^ Interestingly, some differences in the 25^th^, 50^th^, and 75^th^percentiles were found, showing that we cannot extrapolate data from one population to another. Therefore, initiatives to establish ASCVD risk in percentiles of our own population seem truly important.
